# Potential Role of Protein Kinase C in the Pathophysiology of Diabetes-Associated Atherosclerosis

**DOI:** 10.3389/fphar.2021.716332

**Published:** 2021-07-02

**Authors:** Chih-Feng Lien, Sy-Jou Chen, Min-Chien Tsai, Chin-Sheng Lin

**Affiliations:** ^1^ Division of Cardiology, Department of Medicine, Tri-Service General Hospital, National Defense Medical Center, Taipei, Taiwan; ^2^ Department of Emergency Medicine, Tri-Service General Hospital, National Defense Medical Center, Taipei, Taiwan; ^3^ Department of Physiology and Biophysics, Graduate Institute of Physiology, National Defense Medical Center, Taipei, Taiwan

**Keywords:** PKC, atherosclerosis, diabetes, hyperglycemia, inflammation, plaque evolution

## Abstract

Diabetes mellitus is a metabolic syndrome that affects millions of people worldwide. Recent studies have demonstrated that protein kinase C (PKC) activation plays an important role in hyperglycemia-induced atherosclerosis. PKC activation is involved in several cellular responses such as the expression of various growth factors, activation of signaling pathways, and enhancement of oxidative stress in hyperglycemia. However, the role of PKC activation in pro-atherogenic and anti-atherogenic mechanisms remains controversial, especially under hyperglycemic condition. In this review, we discuss the role of different PKC isoforms in lipid regulation, oxidative stress, inflammatory response, and apoptosis. These intracellular events are linked to the pathogenesis of atherosclerosis in diabetes. PKC deletion or treatment with PKC inhibitors has been studied in the regulation of atherosclerotic plaque formation and evolution. Furthermore, some preclinical and clinical studies have indicated that PKCβ and PKCδ are potential targets for the treatment of diabetic vascular complications. The current review summarizes these multiple signaling pathways and cellular responses regulated by PKC activation and the potential therapeutic targets of PKC in diabetic complications.

## Introduction

Diabetes mellitus (DM), a highly prevalent disease worldwide, is caused by insufficient insulin production or insulin resistance. According to the International Diabetes Federation, the global prevalence rate of diabetes in 2019 was 9.3%, which is equivalent to 463 million people. The prevalence is estimated to have increased to 10.2% (578 million people) by 2030 and 10.9% (700 million people) by 2045 (Saeedi et al., 2019). The development of diabetes begins in the prediabetic stage, wherein the fasting plasma glucose levels are elevated due to peripheral resistance to insulin. This stage is characterized by increased insulin secretion by β cells, resulting in hyperinsulinemia. However, long-term overproduction of insulin induces β cell failure, which results in progressive hyperglycemia (Bar-Tana, 2020). Once blood glucose levels exceed the normal levels, patients with diabetes rapidly develop complications, such as kidney, nerve, eye, and cardiovascular diseases. The leading cause of death in diabetic patients is macro- and microvascular complications. Atherosclerosis is one of the main complications of diabetes and is a major contributor to cardiovascular morbidity and mortality (La Sala et al., 2019). Hyperglycemia, hypertension, and hyperlipidemia often occur in patients with type 2 diabetes. These metabolic syndromes damage blood vessels and further induce vasculopathy and accelerate the progress of atherosclerosis, resulting in myocardial infarction and stroke (Colwell et al., 1981; Cooper et al., 2001).

**FIGURE 1:**
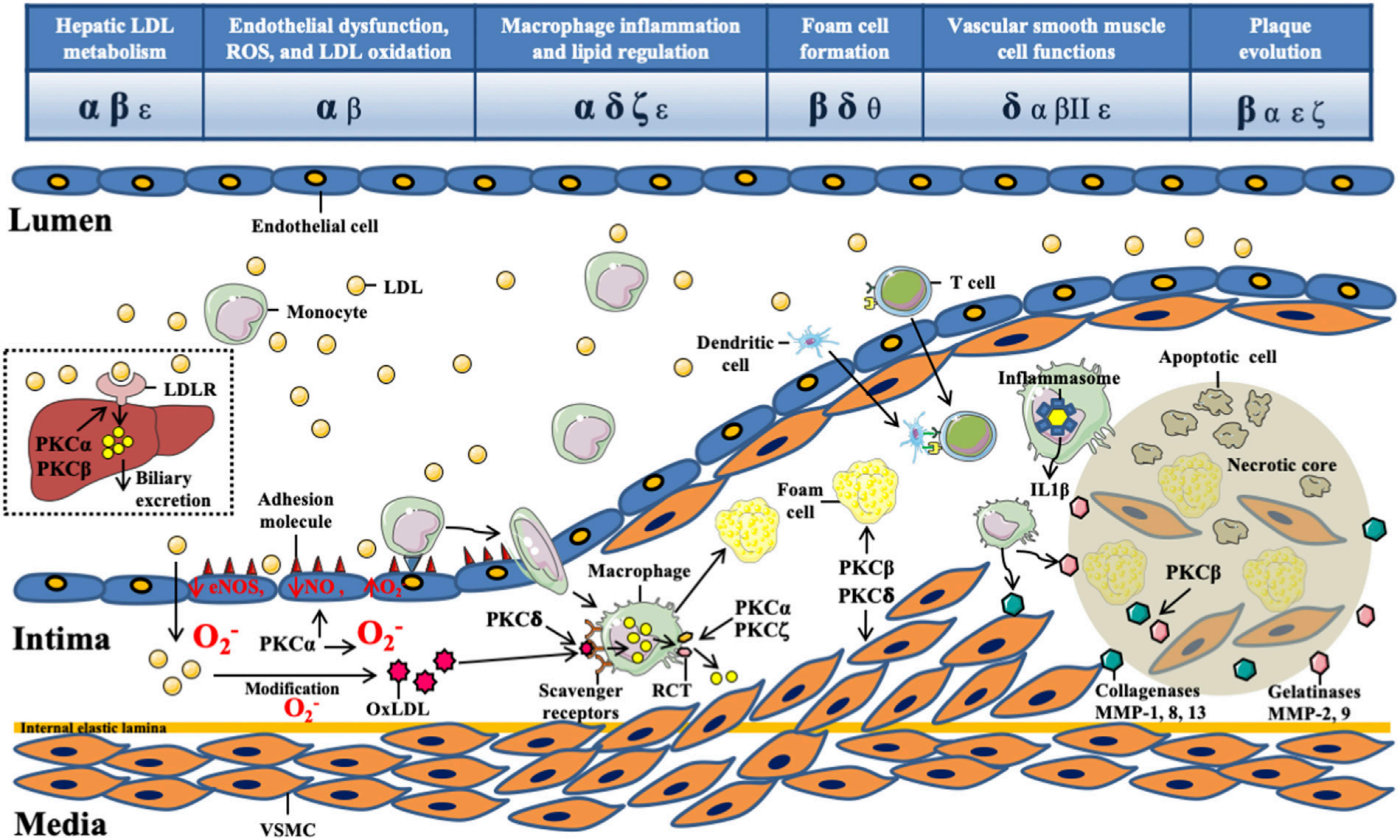
The role of protein kinase C isoforms in the initiation and progression of atherosclerosis. The pathophysiology of atherosclerosis begins with the retention of circulating low-density lipoproteins (LDL) in the intima. Excess levels of circulating LDL are cleared via hepatic LDL receptor, which is regulated by protein kinase Cα (PKCα) and PKCβ. The intimal LDLs are prone to oxidative modification by environmental oxidizing enzymes to become oxidized LDLs (oxLDLs), which activate endothelial cells (ECs) to express adhesion molecules. The major PKC isoforms involved in this step are PKCα and PKCβ. PKCα, PKCδ, and PKCζ regulate the expression of ATP-binding cassette transporter A1, which plays a key role in macrophage cholesterol efflux. PKCβ and PKCδ regulate foam cell formation via manipulating scavenger receptor-mediated uptake of oxLDLs by macrophages. Vascular smooth muscle cells (VSMCs) proliferate and migrate from the media to the intima in response to environmental stimuli. PKCδ plays a critical role in regulating VSMC function during atherosclerosis. Both innate and adaptive immune systems are involved in the atherosclerotic process. Apoptosis of VSMCs and secretion of matrix metalloproteinases (MMPs) from macrophages are critical for plaque complexity. PKCδ is the principal isoform regulating VSMC apoptosis. Finally, PKCβII had been proposed to regulate MMP secretion in ECs during atherosclerosis.

Atherosclerosis is a complex disease involving the interplay of various cell types, such as endothelial cells (ECs), vascular smooth muscle cells (VSMCs), macrophages, and other immune cells (Weber and Noels, 2011). ECs produce several inflammatory mediators, such as CCL5 and CXCL4, and express leukocyte adhesion molecules, such as E-selectin, P-selectin, intercellular adhesion molecule-1 (ICAM-1), and vascular cell adhesion molecule-1 (VCAM-1), which attract monocyte migration and infiltration into the subendothelial space (Weber and Noels, 2011). Subsequently, foam cell formation from monocyte-derived macrophages involves the uptake of low-density lipoprotein (LDL) or oxidize LDL (oxLDL). OxLDL and foam cells accumulate in the vascular intima, which induces oxidative stress and releases platelet-derived growth factor to trigger the VSMC phenotype switch. The phenotypic switching of VSMCs contributes to necrotic core formation and inflammation, and produces extracellular matrix in the fibrous cap (Basatemur et al., 2019). Collectively, the aforementioned cellular processes of ECs, VSMCs, and macrophages play crucial roles in the pathogenesis of atherosclerosis (Figure 1).

Recent studies have demonstrated that three important factors of hyperglycemia promote atherosclerosis, consisting of advanced glycation end products (AGE), oxidative stress, and activation of protein kinase C (PKC) (Aronson and Rayfield, 2002; Yuan et al., 2019). AGEs are products of non-enzymatic glycation and oxidation of proteins and lipids that accumulate in diabetes. AGEs bind to the receptor for AGEs (RAGEs) involved in scavenger receptor A (SR-A) to stimulate reactive oxygen species (ROS) production and promote the progression of pathogenic inflammation in atherosclerosis (Yuan et al., 2019). Hyperglycemia activates the polyol and hexosamine pathways to increase biochemical reactions and further increase ROS production (Paul et al., 2020). PKC mediates the anti-inflammatory effects of cannabidiol and 17β-estradiol against high glucose-induced arrhythmia, which could be a cardioprotective therapeutic approach in diabetic complications (Fouda and Ruben, 2021). Moreover, hyperglycemic activation of PKC signaling leads to increased endothelial inflammation, endothelial relaxation dysfunction, and foam cell formation, which induces the expression of permeability-enhancing factor vascular endothelial growth factor (VEGF) and decreases nitric oxide (NO) generation in VSMCs (Rask-Madsen et al., 2010; Yuan et al., 2019). All the results emphasize the unique characteristics of diabetes-associated atherosclerosis.

Several *in vivo* and human studies had pointed out the intriguing presentations of diabetes-associated atherosclerosis. A key regulator, the RAGE gene, contributes to the diabetes-induced inflammation with control of cholesterol efflux, monocyte recruitment, macrophage infiltration, and lipid content in diabetic plaque (Daffu et al., 2015; Senatus et al., 2020). Importantly, study using internal mammary arteries collected from diabetic patients revealed more pronounced endothelial dysfunction with impaired relaxation in response to EC stimulated with acetylcholine and reduced eNOS expression and activity compared with those from non-diabetic patients (Okon et al., 2005). Additionally, clinical studies demonstrated that there are higher contents of necrotic core, lipid content, macrophage infiltration, RAGE, matrix metalloproteinase 2 (MMP2), MMP9, thinner fibrous cap, and healed cap rupture in the plaque area in diabetic patients than those in non-diabetic patients (Cipollone et al., 2003; Burke et al., 2004; Virmani et al., 2006; Sugiyama et al., 2018). These results highlight the human evidence of diabetes-accelerated atherosclerosis and plaque instability. This review summarized the essential role of PKC in diabetes and atherosclerosis, providing therapeutic targets for diabetes-associated atherosclerotic cardiovascular disease (ASCVD).

## Protein Kinase C Family

PKC enzymes were initially identified as nucleotide-independent, calcium-dependent serine kinases (Inoue et al., 1977). These enzymes contain a highly conserved catalytic (kinase) domain and have more variable regulatory domains. At least 11 isoforms of PKC have been identified, which can be further divided into three subfamilies based on their NH_2_-terminal regulatory domain structure and cofactor regulation: classical, novel, and atypical (Steinberg, 2008). The classical PKCs (α, βI, βII, and γ) contain tandem diacylglycerol (DAG)-binding C1 domains and a phosphatidylserine- and calcium-binding C2 domains in their regulatory domain, and are dependent on DAG and calcium for their activity. The novel PKCs (δ, ε, θ, and η) contain a C2 domain that lacks critical calcium-coordinating acidic residues, and thus, they require DAG, but not calcium, for their activity. The atypical PKCs (ζ, ι/λ), which lack a calcium-sensitive C2 domain and contain only one atypical C1 domain, are unresponsive to DAG and calcium; however, they are activated by other lipid-derived second messengers (Steinberg, 2008). Upon activation, the PKCs translocate from the cytosol to the plasma membrane and other cellular organelles, where they undergo a conformational change, expel the autoinhibitory pseudosubstrate domain from the substrate-binding pocket, activate phosphorylation of their substrates, and elicit biological actions (Steinberg, 2008). In addition to diabetes and atherosclerosis, PKC regulates several cellular functions, including cell death and proliferation, gene transcription and translation, alteration of cell shape and migration, regulation of cell-cell contact and secretion, and regulation of ion channels and receptors, which is involved in several diseases, such as cancer, heart failure, stroke, neurodegenerative diseases, autoimmune diseases, and psychiatric disorders (Mochly-Rosen et al., 2012). Such evidence points out the crucial role of PKC in disease development.

## The Role of Protein Kinase C in Atherosclerosis

### Protein Kinase C Regulates Hepatic LDL Receptor Expression

The liver is the major organ for cholesterol synthesis and degradation. LDL accumulation in the subendothelial space is the initial stage of atherosclerosis. Therefore, the removal of LDLs from the blood *via* regulation of the hepatic LDL receptor (LDLR) plays a vital role in atherosclerosis therapy. A human study revealed that young patients with type 1 or 2 diabetes have a high prevalence of lipoprotein abnormalities. Poor glycemic control increases the prevalence of these lipoprotein risk factors (Albers et al., 2008). Obese humans express high levels of *PRKCD*, which is positively correlated with fasting glucose and circulating triglycerides (Bezy et al., 2011). In particular, deletion of the *PRKCD* gene revealed increased hepatic insulin signaling and reduced expression of gluconeogenic and lipogenic enzymes. Previous studies demonstrated that the PKC activator phorbol 12-myristate 13-acetate (PMA) promotes LDLR gene expression *via* SREBP2 activation (Wong et al., 2016). Furthermore, PMA-activated PKCα, PKCβ, and PKCε increased LDLR gene transcription and mRNA stability through extracellular signal-regulated kinase (ERK) and c-Jun N-terminal kinase (JNK) signaling pathways (Kumar et al., 1997; Mehta et al., 2002; Kapoor et al., 2003; Vargas et al., 2009). Study from Huang et al. (2004) indicated that PKCβ and PKCε may regulate LDLR transcription through hyperphosphorylation of histone H3-Ser10 at the LDLR promoter. In addition, animal studies have indicated that treatment of rats with 17-β-oestradiol activates PKCα and upregulates LDLR expression in the liver (Marino et al., 2001). Collectively, these studies suggest that PKC isoforms, namely PKCα, PKCβ, PKCε, and PKCδ, contribute to cholesterol regulation in hepatocytes. Table 1 summarizes the current understanding of the involvement of each PKC isoform in atherosclerosis.

**TABLE 1:** Summary of PKC isoform-specific role in the progress of atherosclerosis.

Atherosclerosis etiology	PKC isoform	Stimulus/intervention	Working model	Effect of specific PKC isoform	References
Hepatic LDLR expression	PKC	Phorbol esters	HepG2	Upregulation and stabilization of LDLR mRNA	Vargas et al. (2009)
	PKCα	Phorbol esters	HepG2	Increases LDLR protein expression	Kumar et al. (1997)
	PKCα	17-β-oestradiol	Rats	Upregulation of LDLR in liver, PKCα activation	Marino et al. (2001)
	PKCβ	Phorbol esters	HepG2	Upregulation of LDLR mRNA and protein expression	Wong et al. (2016)
	PKCβ	Overexpression	HepG2	Upregulation of LDLR protein expression	Kapoor et al. (2003)
	PKCβ	Phorbol esters	HepG2	Increases LDLR promoter activity	Huang et al. (2004)
	PKCε	Cholesterol depletion, overexpression, AS	HepG2	Increases LDLR promoter activity	Mehta et al. (2002)
	PKCε	Phorbol ester	HepG2	Increases LDLR promoter activity	Huang et al. (2004)
Endothelial dysfunction	PKC	OxLDL	HUVECs	Increases ICAM-1, VCAM-1, MCP-1, E-selectin, CCR2, CXCL2 mRNA expression	(Sun et al., 2018; Zhang et al., 2019)
	PKCα	OxLDL	HUVECs	Enhances production of eNOS-derived superoxide anion	Fleming et al. (2005)
	PKCα	Phorbol esters, thymeleatoxin	Human ECs	Increases arginase expression and activity, decrease NO production	Visigalli et al. (2010)
	PKCα	Inhibition of mTOR	HAECs	Decreases inflammation/PKCα activation	Fan et al. (2019)
	PKCβ	Deletion of PKCβ	Mice	Increases MMP2, VCAM-1 protein expression	Harja et al. (2009)
Macrophage inflammation and lipid regulation	PKCα	Phorbol esters/PKC inhibitor	Raw264.7	Stabilizes ABCG1 and increases cholesterol efflux	Watanabe et al. (2019)
	PKCε	Resistin	PBMC	Increases macrophages inflammation	Zuniga et al. (2017)
	PKCζ	Overexpression Hsp27	THP-1	Increases ABCA1 expression and cholesterol efflux	Kuang et al. (2017)
	PKCδ	Linoleic acid/siRNA	Raw264.7	Destabilizes ABCA1	Wang and Oram, (2007)
	PKCδ	OxLDL, HFD	BMDM, mice	Decreases macrophage apoptosis, increases proliferation and inflammation	Li et al. (2017)
	PKCδ	OxLDL/shRNA, siRNA, rottlerin	THP-1, PBMC, BMDM	Increases oxLDL uptake	Szilagyi et al. (2014)
Foam cell formation	PKCβ	OxLDL/PKCβ inhibitor	THP-1, PBMC	Increases SR-A expression, oxLDL uptake and foam cell formation	Osto et al. (2008)
	PKCθ	Thrombin HFD	Raw264.7, peritoneal macrophages	Increases CD36 expression, and foam cell formation	Raghavan et al. (2018)
	PKCδ	OxLDL/shRNA, siRNA, rottlerin	THP-1, HMDMs	Increases SR-A, CD36 expression, oxLDL uptake and foam cell formation	Lin et al. (2012)
Vascular smooth muscle cells functions	PKCδ	Overexpression	Rat VSMCs	Reduces proliferation of VSMCs	Fukumoto et al. (1997)
	PKCδ	PKCδ knockout	Mice	Promotes apoptosis of VSMCs and reduces vein graft atherosclerosis	Leitges et al. (2001)
	PKCδ	OxLDL	Human primary VSMC	Promotes ROS production and ER stress-induced apoptosis	Larroque-Cardoso et al. (2013)
	PKCδ	Rottlerin, siRNA	Rat aortic SMC	Mediates oxidative stress-induced apoptosis	Kato et al. (2009)
	PKCδ	Rottlerin	Rat VSMCs	Regulates VSMC proliferation	Ginnan et al. (2004)
	PKCδ	Ang II/siRNA, rottlerin	Rat VSMCs	Activates smooth muscle 22α and p47^*phox*^ to generate ROS	Lv et al. (2012)
	PKCδ	PDGF and mechanical stress/siRNA	Mice aorta VSMCs	Increases migration of VSMCs	Li et al. (2003a)
	PKCα/ε	ROS/PKC inhibitor	Rat VSMCs	Induces apoptosis of VSMCs/PKCα and PKCε activation	Li et al. (1999)
	PKCα/βII	Serum starvation/overexpression	Rat VSMCs	Inhibition of apoptosis of VSMCs	Hall et al. (2000)
	PKCβI	oxLDL phorbol esters/RBX, icariin		Increases proliferation and migration of VSMCs	Zhang et al. (2021)
	PKCβII	Denudation injury, TNFα/PKCβ Tg	Mice arteries or VSMCs	Increases migration of VSMCs and neointimal expansion	Huang et al. (2010)
	PKCε	PDGF/shRNA	Mice VSMCs	Induces migration of VSMCs	Quintavalle et al. (2010)
Plaque evolution	PKCβ	OxLDL	HCAECs	Increases MMP-1 and MMP-3 expression	Li et al. (2003b)
	PKCα/βI	IL-1β/PKC inhibitor	Human ECs	Increases MMP-2 expression	Mountain et al. (2007)
	PKCβ	OxLDL/PKCβ inhibitor	Human and mice aortic ECs	Increases MMP-2 expression	Harja et al. (2009)
	PKCβ	PKCβ gene knockout	ApoE^−/−^ mice	Increases MMP-2 expression and atherosclerotic lesion size and complexity	Harja et al. (2009)
	PKCβII	Denudation injury, TNFα/PKCβ Tg	Mice arteries or VSMCs	Increases MMP-9 expression and secretion	Huang et al. (2010)
	PKCζ	bFGF and IL-1/AS, DN mutation	Rabbit VSMCs	Increases MMP-1, -3, -9 secretion	Hussain et al. (2002)
	PKCε	Resistin/PKCε inhibitor	Human coronary VSMCs	Increases MMP-2, -9 expression	Ding et al. (2011)

LDLR, low density lipoprotein receptor; AS, antisense oligonucleotide; HUVECs, human umbilical vein endothelial cells; ICAM-1, intercellular cell adhesion molecule-1; VCAM-1, vascular cell adhesion molecule-1; MCP-1, monocyte chemoattractant protein-1; CCR2, C-C chemokine receptor type 2; CXCL2, C-X-C Motif Chemokine Ligand 2; HAECs, human aortic endothelial cells; MMP, matrix metalloproteinase; ABCA1, ATP binding cassette subfamily A member 1; ABCG1, ATP binding cassette subfamily G member 1; HFD, High-fat diet; BMDMs, bone marrow-derived macrophages; PBMC, peripheral blood mononuclear cell; HMDMs, human monocyte-derive macrophages; HCAECs, human coronary artery endothelial cells; PDGF, platelet-derived growth factor; DN, dominant negative; Tg, transgenic inhibition; ROS, Reactive oxygen species; PKC, protein kinase C; IL, interleukin; Ang II, Angiotensin II, RBX, ruboxistaurin.

### Mutual Interaction Between Reactive Oxygen Species and Protein Kinase C

Oxidative stress results from excessive ROS production mainly from mitochondria and NADPH oxidases. Other enzymes, such as xanthine oxidase, NO synthase, and lipoxygenase, also generate ROS (Holmstrom and Finkel, 2014). PKC-dependent activation of nicotinamide adenine dinucleotide phosphate (NADPH) oxidase is considered to be one of the major sources for high glucose-induced ROS overproduction. Hyperglycemia increases intracellular DAG concentration, leading to PKC activation which enhances NADPH oxidase by promotes p47^*phox*^ translocation from cytosol to membrane (Inoguchi et al., 2000; Yuan et al., 2019). In addition, metformin or liraglutide inhibits hyperglycemia-induced ROS production though downregulation of PKC-dependent NADPH oxidase in human ECs (Batchuluun et al., 2014), which partly elucidates their athero-protective effects. Interestingly, hyperglycemia increases mitochondrial superoxide production, leading to PKC activation (Nishikawa et al., 2000). ROS activates PKC by generation of lipid cofactors and regulation of calcium levels. Moreover, direct modification of cysteine residues in PKCs and tyrosine phosphorylation of PKCδ by oxidative stress result in activation of PKC (Steinberg, 2015). Undoubtedly, ROS and PKC mutually interact to form a vicious circle during atherosclerosis.

### Protein Kinase C and Endothelial Dysfunction

The endothelium is the main regulator of vascular wall homeostasis. ECs produce NO by eNOS to maintain vascular tone and regulate leukocyte infiltration (Tousoulis et al., 2012). In the early stage of atherosclerosis, oxLDL inhibits eNOS activation to reduce NO production (Hirata et al., 1991), leading to increased expression of adhesion molecules, increased synthesis of pro-inflammatory and pro-thrombotic factors, increased oxidative stress, and abnormal modulation of vascular tone (Sitia et al., 2010). PKCα has been demonstrated to play an important role in endothelial dysfunction. Human umbilical vein endothelial cells (HUVECs) treated with oxLDL reduced PKCα activity, which is associated with dephosphorylation of eNOS at Thr495, dissociation of the eNOS signaling complex, and enhanced production of eNOS-derived superoxide anion (Fleming et al., 2005). In human ECs, activation of PKC by phorbol esters or thymeleatoxin stimulates entry of arginine via induction of cationic amino acid transporters 2 (CAT2) arginine transporters, and increases arginase expression and activity, which shifts arginine metabolism from NO synthesis to ornithine and urea production, thereby promoting eNOS phosphorylation at Thr495 and reducing NO production. These effects, possibly mediated by PKCα, require activation of the MEK/ERK1/2 cascade, which stimulates activator protein-1 activity and CAT2 expression (Visigalli et al., 2010). Furthermore, oxLDL stimulated endothelin-1 (ET-1) and mechanistic target of rapamycin 2 (mTOR2) expression to induce the expression of adhesion molecules, such as ICAM-1, VCAM-1, and E-selectin, through the PKC signaling pathway (Sun et al., 2018; Zhang et al., 2019). Inhibition of mTOR enhances PKCα phosphorylation and increases the level of miR-200a-3p, which reduces TNFα-induced VCAM-1 expression and monocytic cell adhesion (Fan et al., 2019). In addition to PKCα, PKCβ regulates VCAM-1 and MMP2 expression in oxLDL-treated human aortic ECs, which promotes atherogenesis (Harja et al., 2009). All these studies point out that PKC isoforms contribute to endothelial dysfunction by regulating NO production and inflammatory responses in hypercholesterolemia and hyperglycemia.

### Protein Kinase C in Macrophage Inflammation and Lipid Regulation

The formation of foam cells involves the uptake of LDL and modified LDL, and the efflux of excess cholesterol by macrophages (Kunjathoor et al., 2002; Kruth et al., 2005; Tall, 2008; Moore and Tabas, 2011). LDL uptake occurs through several mechanisms, including fluid-phase pinocytosis of native LDL (Kruth et al., 2005). Substantial evidence suggests that oxLDL uptake by macrophages can occur *via* SRs, namely SR-A, CD36, and lectin-like oxidized low-density lipoprotein receptor-1 (Kunjathoor et al., 2002; Moore and Freeman, 2006). Recent work on the regulation of foam cells has also focused on cholesterol efflux from tissues, which leads to regression of atherosclerotic lesions (Tall, 2008). Reverse cholesterol transporters, such as ATP binding cassette subfamily A member 1 (ABCA1) and ABCG1, are thought to mediate more than 70% of total cholesterol efflux from macrophage foam cells after cholesterol loading (Tall, 2008).

PKC isoforms involve several signaling pathways that contribute to different roles in macrophages during atherogenesis. Previous studies showed that the high levels of plasma resistin in patients with ASCVD are associated with high levels of inflammatory cytokines. Resistin upregulating inflammatory cytokine gene expressions of CD40L, interleukin (IL)-12p40, IL-6, TNF-α, MIP-1α, and MIP-1β is mediated by PKCε and TLR4 in human macrophages (Zuniga et al., 2017).

Activation of PKCα results in phosphorylation of ABCG1 and prevention ABCG1 from degradation, which facilitates cholesterol efflux from cells (Watanabe et al., 2019). Moreover, PKCζ-mediated hsp27-enhanced ABCA1 expression and cholesterol efflux in THP-1 macrophages (Kuang et al., 2017). Interestingly, unsaturated fatty acids phosphorylate and destabilize ABCA1 through PKCδ signaling pathway, but not through PKCβ and PKCθ signaling pathways, in murine RAW 264.7 macrophages (Wang and Oram, 2007). All these results point out the diverse effects of specific PKC isoform in regulation of cholesterol efflux.

Studies using pharmacological inhibitors demonstrate that inhibition of PKCβ prevents oxLDL uptake in human macrophages by reducing SR-A expression (Osto et al., 2008). Moreover, thrombin inducing CD36 expression and foam cell formation is through Gα12-Pyk2-Gab1-PKCθ-dependent ATF2 activation, which contributes to diet-induced atherogenesis (Raghavan et al., 2018). We previously demonstrated that knockdown of PKCδ by shRNA or siRNA effectively reduced SR-A, CD36 expression, oxLDL uptake, and foam cell formation through PI3K/Akt and ERK signaling pathways in THP-1-derived macrophages and human primary macrophages (Lin et al., 2012). The highly expressed PKCδ in human atherosclerotic arteries and infiltrating CD68-positive macrophages further emphasized the role of PKCδ on foam cell formation during atherosclerosis. However, study from Szilagyi et al. (2014) indicated that inhibition of PKCδ did not change CD36 expressions and foam cell formation in human monocyte-derived macrophages (HMDMs) from healthy donors and BMDMs from PKCδ KO mice. Intriguingly, in diabetic rats, the protein levels of PKCδ are increased in macrophages (Li et al., 2017). Depletion of PKCδ promotes atherosclerotic plaque formation in *ApoE* KO mice. However, the protein expression of CD36 and acetylated LDL uptake by peritoneal macrophages did not differ between PKCδ/*ApoE* KO and *ApoE* KO mice. Further confirmatory studies are necessary to prove the role of PKCδ on foam cell formation and atherosclerosis.

### Protein Kinase C Regulates Vascular Smooth Muscle Cells Functions

In response to environmental cues and atherogenic stimuli, such as lipids, lipoproteins, ROS, and inflammatory mediators, VSMCs can shift from a contractile phenotype to a synthetic phenotype, namely SMC phenotypic switching (Allahverdian et al., 2018). The contractile phenotype is characterized by high levels of contractile gene expression, such as α-smooth muscle actin (αSMA), smooth muscle 22α (SM22α), and heavy-caldesmon (h-Cad), and low rates of proliferation, migration, and extracellular matrix production. In contrast, the synthetic phenotype is characterized by reduced contractile gene expression and increased rates of proliferation, migration, and extracellular matrix production (Owens et al., 2004; Allahverdian et al., 2018). Moreover, apoptosis of VSMCs may occur due to cyclic stretch of proliferated VSMCs and continuous uptake of oxLDL, which is possibly mediated by PKC (Li et al., 2000). The subsequent effect of VSMCs on atherosclerosis is complex because both proliferation and apoptosis of VSMCs coincide with atherosclerotic lesions. Although apoptosis of VSMCs can reduce intimal hyperplasia, which promotes early atherosclerotic lesions, the accumulation of apoptotic VSMCs may contribute to the instability and rupture of advanced plaques (Weissberg et al., 1996; Leitges et al., 2001).

Several studies proposed the role of PKCδ on apoptosis. Studies using *Prkcd* null mice suggested that vein graft arteriosclerosis is accelerated due to the resistance of PKCδ^−/−^ VSMCs to apoptosis compared that of VSMCs from wild-type mice (Leitges et al., 2001). PKCδ contributes to oxLDL-induced endoplasmic reticulum (ER) stress and apoptosis in human VSMCs (Larroque-Cardoso et al., 2013). Additionally, caspase-3-mediated PKCδ cleavage is mandatory for oxidative stress-induced VSMC apoptosis, and PKCδ acts both upstream and downstream of caspase-3 (Kato et al., 2009). Such evidence highlights the crucial roles PKCδ of VSMC apoptosis.

Additionally, overexpression of PKCδ reduces proliferation of VSMCs by arresting cells in the G1 phase and inhibiting the expression of cyclin D1 and cyclin E (Fukumoto et al., 1997). However, PKCδ is involved in the regulation of VSMC proliferation through ERK signaling, which is a major regulatory pathway for cell growth and proliferation (Ginnan et al., 2004). PKCδ mediates angiotensin II-stimulated ROS generation and proliferation of VSMCs via interaction with smooth muscle 22α and p47^*phox*^ (Lv et al., 2012). These results revealed the controversial roles of PKCδ on VSMC proliferation. Additionally, PKCδ is activated and translocated to the cytoskeleton during VSMC migration under mechanical stress. VSMCs from *Prkcd* null mice showed reduced migration in response to platelet-derived growth factor and mechanical stress (Li C. et al., 2003). Corroborating the finding that PKCδ is one of the major PKC isoforms expressed in VSMCs (Fukumoto et al., 1997), these studies provide evidence for the important roles of PKCδ in VSMC-mediated atherosclerosis.

Although PKCδ plays a major role in regulating VSMC functions, other PKC isoforms might contribute to ROS-induced apoptosis of VSMCs, including PKCα, PKCβII, and PKCε (Li et al., 1999). Overexpression of PKCα or PKCβII, but not PKCδ, reduces serum starvation-induced apoptosis in VSMCs (Hall et al., 2000). Icariin suppresses PKCβI expression and inhibits oxLDL-induced cell proliferation and migration in human aortic SMCs and exhibits athero-protective effects in *ApoE* null mice (Zhang et al., 2021). *In vivo* transgenic inhibition of PKCβII suppresses VSMC proliferation and neointimal expansion in response to acute artery injury, possibly *via* regulation of the ERK and Erg-1 signaling pathways (Huang et al., 2010). Moreover, silencing of PKCε reduces VSMC migration (Quintavalle et al., 2010). These results suggest that PKC isoforms other than PKCδ might regulate VSMC proliferation, apoptosis, and migration during atherosclerosis.

### Protein Kinase C in the Progress of Plaque Complexity

Although atherosclerotic plaques cause lumen narrowing and myocardial tissue ischemia, the culprit lesions from patients who died of acute atherothrombotic events are characterized not by their size, but by several distinct morphological features, such as large necrotic cores, a ruptured or eroded fibrous cap, and an occlusive thrombus (Tabas, 2011). Vulnerable plaques with large necrotic cores and thin fibrous caps are highly predictive of progression to culprit lesions and are usually observed in patients with acute coronary syndrome (Tabas, 2011). Thinning of fibrous caps in vulnerable plaques is mainly due to apoptosis of VSMCs and the secretion of MMPs from vascular cells, particularly collagenases (MMP-1, MMP-8, and MMP-13) for the initial proteolytic cleavage of the intact collagen fibrils and gelatinases (MMP-2 and MMP-9) for further degradation of the cleaved collagen (Clarke et al., 2006; Libby, 2008). The second critical feature of vulnerable plaques is plaque necrosis. In early plaques, VSMCs and macrophages undergo apoptosis, and the dead cells are rapidly removed by neighboring phagocytes (Tabas, 2010; Moore and Tabas, 2011; Tabas, 2011). However, such efferocytosis becomes less efficient under prolonged ER stress conditions in advanced lesions, and the plaques then develop necrotic cores, which further destabilize the plaques (Tabas, 2010; Moore and Tabas, 2011; Tabas, 2011). Therefore, recent studies of plaque evolution have primarily focused on ER stress-induced apoptosis of macrophages and defective efferocytosis, apoptosis of VSMCs, and secretion of MMPs by macrophages (Clarke et al., 2006; Libby, 2008; Tabas, 2010).

Non-selective chemical inhibition of PKC has no effect on ER stress-induced apoptosis of macrophages, suggesting that PKC is not involved in this process (Seimon et al., 2010). However, several studies have shown that PKC is involved in MMP expression in vascular cells. OxLDL activates PKC and increases MMP-1 and MMP-3 expression in human coronary artery ECs, which is blocked by inhibition of PKC (Li et al., 2003). Further studies have shown that inhibition of PKCα/βI reduces IL-1β-induced MMP-2 expression in human ECs (Mountain et al., 2007). Moreover, inhibition of a PKCβ isoform, namely PKCβII, decreases oxLDL-induced MMP-2 expression in primary murine and human aortic ECs (Harja et al., 2009). Transgenic inhibition of PKCβII suppresses TNFα-induced MMP-9 expression in VSMCs and reduces denudation-induced MMP-9 expression in mouse femoral arteries, possibly *via* regulation of ERK- and Erg-1-dependent signaling (Huang et al., 2010). The role of PKCβII in MMP expression and secretion has also been examined in atherosclerosis-prone *ApoE* null mice. PKCβII, but not PKCβI, was activated in the aortas of *ApoE* null mice. Chemical inhibition of PKCβ or genetic deletion of PKCβ decreases MMP-2 expression in the aorta, which reduces the complexity of atherosclerotic lesions (Harja et al., 2009). PKCζ, in addition to PKCβII, regulates MMP secretion induced by basic fibroblast growth factor and IL-1 in rabbit VSMCs, possibly *via* NFκB signaling (Hussain et al., 2002). Inhibition of PKCε suppresses resistin-induced MMP-2 and MMP-9 expression, resulting in reduced migration of VSMCs (Ding et al., 2011). These studies highlight the important roles played by PKCβ, PKCε, and PKCζ in regulating MMP expression during atherosclerotic plaque evolution.

## The Role of Protein Kinase C in Diabetes-Associated Atherosclerosis

**FIGURE 2:**
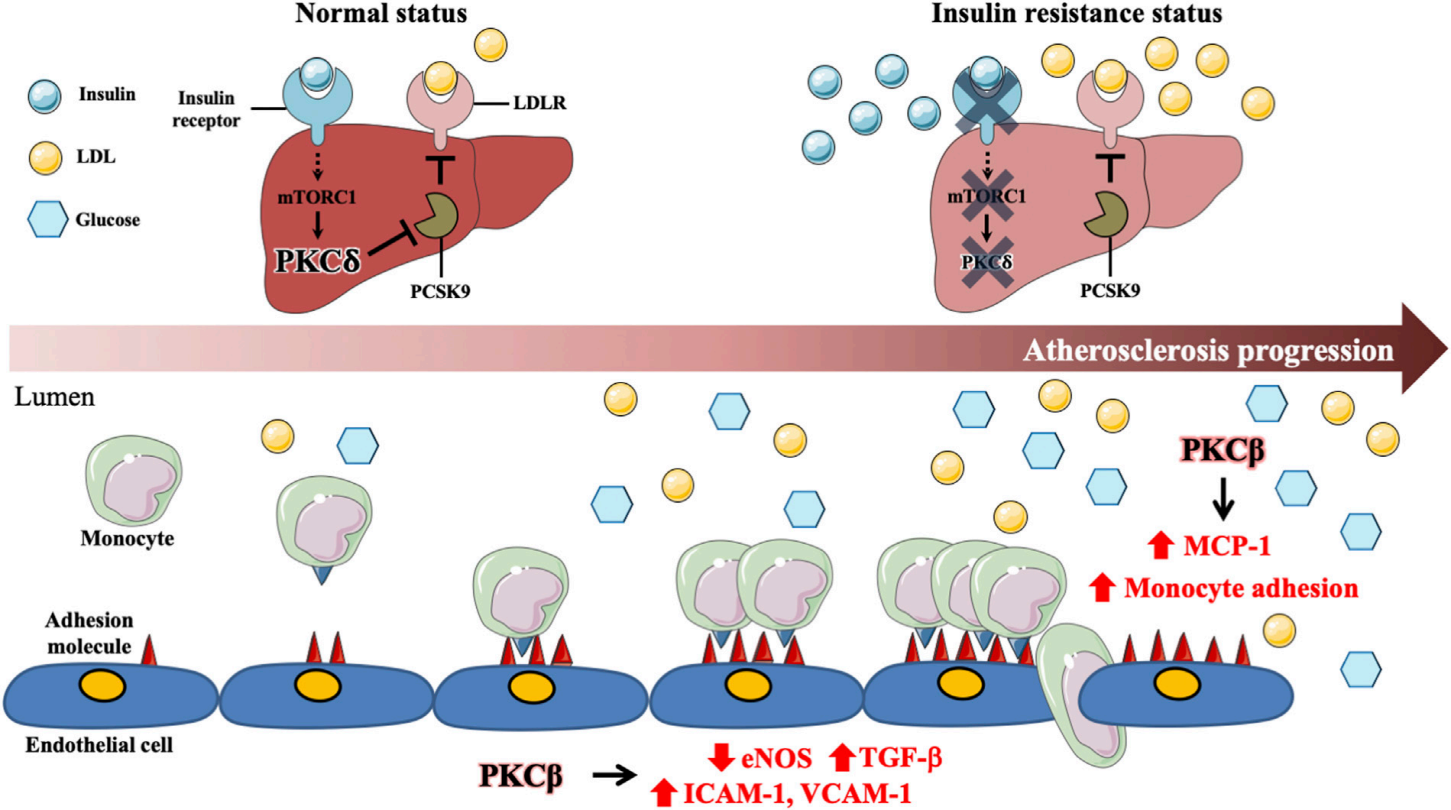
Involvement of specific protein kinase C isoform in diabetes-associated atherosclerosis. In the normal status, activated insulin signaling enhances PKCδ through mTORC1 signaling pathway in hepatocytes. Activated PKCδ maintains LDLR protein expression by inhibiting expression of proprotein convertase subtilisin/kexin type 9 (PCSK9). Under insulin resistance status, impaired insulin signaling results in suppression of mTORC1 and PKCδ expressions, which enhances PCSK9 expression and promotes LDLR degradation, contributing to diabetes-associated dyslipidemia. Hyperglycemia activates PKCβ to decrease eNOS activity and increase TGFβ expression, which enhances adhesion molecules, such as ICAM-1 and VCAM-1, expression in ECs. Moreover, Hyperglycemia further promotes macrophage inflammation and adhesion to ECs *via* PKCβ activation. Those data reveal that PKCδ and PKCβ play central roles in diabetes-associated atherosclerosis.

PKC plays a major role in diabetes and atherosclerosis. A previous study indicated that inhibition of hepatic insulin receptor reduces LDLR protein expression, which is possibly due to upregulating the levels of proprotein convertase subtilisin/kexin type 9 (PCSK9) *via* hepatic mTOR1 mediated PKCδ pathway (Ai et al., 2012). Hyperglycemia activates PKCβ to decrease eNOS activity and increase adhesion molecules, such as ICAM-1, VCAM-1, and E-selectin expression, leading to macrophage adhesion to ECs (Avogaro et al., 2008; Xu et al., 2010). Moreover, in diabetic *ApoE* null mice, PKCβ is involved in diabetes-accelerated atherosclerosis *via* regulation of the IL-18/IL-18BP and MAP kinase signaling pathways and promotes VCAM-1 expression, macrophage adhesion, EC dysfunction, aorta and macrophage inflammatory responses (Kong et al., 2013; Durpes et al., 2015). Additionally, inhibition of PKCβ reduces the expression of ICAM-1 and monocyte chemoattractant protein-1 (MCP-1) and decreases the recruitment of macrophages to the kidneys of diabetic rats (Wu et al., 2006). Although RAGE acts as a key role in diabetes-associated atherosclerosis, the studies on the association between RAGE and PKC in atherosclerosis are limited, which requires further investigation. These data indicate that PKCβ and PKCδ contribute to diabetes-accelerated atherosclerosis with the involvement of hepatic cholesterol metabolism, endothelial dysfunction, vascular inflammation, and monocyte recruitment and adhesion. (Figure 2 and Table 2).

**TABLE 2:** Summary of PKC isoform-specific role in diabetes-associated atherosclerosis.

Step of atherosclerosis	PKC isoform	Stimulus/intervention	Working model	Effect of specific PKC isoform	References
Cholesterol metabolism	PKCδ	siRNA	Tsc1-null MEFs	Upregulation of LDLR protein expression	Ai et al. (2012)
Endothelial dysfunction	PKCβ	AGE/LY333531	HUVEC	Increases ICAM-1 expression and monocytes adhesion to ECs, inflammation	Xu et al. (2010)
	PKCβ	STZ, HFD, HG, palmitate, IL-18/RBX	BAECs, THP-1, HAECs, RAW 264.7, DM ApoE^−/−^ mice	Increases VCAM-1 and IL-18 expression and monocytes adhesion to ECs, enhances plaque formation, complexity, and cholesterol content	Durpes et al. (2015)
	PKCβ	STZ/LY333531	Rat	Increases macrophages recruitment and ICAM-1 and MCP-1 protein expression in the kidney	Wu et al. (2006)
	PKCβ	PKCβ gene knockout/RBX	DM ApoE^−/−^ mice, U937	Increases monocytes content, inflammation and MAPK expression, enhances plaque formation	Kong et al. (2013)

LDLR, low density lipoprotein receptor; HUVECs, human umbilical vein endothelial cells; ICAM-1, intercellular cell adhesion molecule-1; VCAM-1, vascular cell adhesion molecule-1; HAECs, human aortic endothelial cells; PBMC, peripheral blood mononuclear cell; PKC, protein kinase C; IL, interleukin; HFD, High-fat diet; STZ, Streptozotocin; BAECs, bovine aorta endothelial cells; MAPK, mitogen-activated protein kinases; RBX, ruboxistaurin.

## Pharmacologic Inhibition of Protein Kinase C in Human Clinical Trials

Based on structural and functional approaches, there are several potential targets for pharmacological development of PKC, including the ATP-binding site in the catalytic region, the DAG-binding site, anchoring protein-binding sites in the C2 domain, protein-protein interactions that regulate the subcellular localization of the PKC enzymes, and substrate-binding sites (Mochly-Rosen et al., 2012). Several clinical trials have been conducted for the treatment of cardiovascular disorders, diabetes, cancer, bipolar disorder, and transplantation rejection (Mochly-Rosen et al., 2012) (ClinicalTrials.gov). Despite the promising effects of PKC modulators in human disease, results in clinical trials remain controversial. A large randomized trail using adenosine, which activates PKCδ and PKCε, during acute myocardial infarction proposed that treatment with high-dose adenosine significantly reduces infarct size compared to placebo (Ross et al., 2005). Intra-coronary administration of delcasertib, a peptide inhibitor of PKCδ, exhibits a trend toward reduced infarct size and promoted resolution of ST elevation (Direct Inhibition of delta-Protein Kinase et al., 2008). Certain clinical trials of ruboxistaurin (RBX), a PKCβ inhibitor, in the treatment of diabetic complications revealed that inhibition of PKCβ significantly reduces diabetic retinopathy, nephropathy, and neuropathy (Geraldes and King, 2010; Mochly-Rosen et al., 2012). Additionally, in a clinical trial of diabetic retinopathy, where 252 patients received a placebo or RBX (8, 16, or 32 mg/day) for 36–46 months, treatment with 32 mg/day RBX delayed the occurrence and reduced the risk of moderate visual loss (MVL) and sustained MVL (SMVL) compared to that with the placebo (Group, 2005). Although the outcome of clinical trials needs further validation, the current review suggests the promising role of PKC in diabetes-associated atherosclerosis.

## Potential Challenges and Future Perspectives

PKC remains an elusive drug target, and no single compound has been identified to specifically target PKC (Mochly-Rosen et al., 2012). Several challenges and limitations need to be overcome before PKC inhibitors can be used to treat atherosclerosis or diabetic atherosclerosis. First, different PKC isoforms may regulate the same target protein with different effects. For instance, 12-O-tetradecanoylphorbol-13-acetate (TPA)-induced phospholipase D1 (PLD1) activation is mediated by PKCα. However, PKCδ overexpression inhibits PLD1 activation. In particular, overexpression of PKCδ inhibits PKCα-mediated PLD1 activation (Oka et al., 2003). Second, different PKC isoforms have different effects in the same disease model. Li et al. (2017) revealed that deletion of PKCδ increases atherosclerotic lesions in *ApoE* null mice, whereas Raghavan et al. (2018) revealed that PKCθ deletion decreases atherosclerotic lesions in *ApoE* null mice. Likewise, PKC isoforms may play a compensatory role in the loss of other PKC isoforms. For instance, deletion of PKCε causes an increase in PKCδ expression and activation in the heart of *Prkce* null mice (Gray et al., 2004). In addition, deletion of PKCθ increases PKCα/βII phosphorylation in peritoneal macrophages isolated from *ApoE* null mice (Raghavan et al., 2018). Third, although several inhibitors of PKC have been developed, the lack of isoform-selectivity in many current compounds is a common problem. Enzastaurin, a PKCβ-selective inhibitor, has been found to inhibit PKC isoforms, namely PKCα, PKCγ, and PKCε (Graff et al., 2005). Moreover, the PKCδ selective inhibitor, rottlerin, has been used in many studies. It has been reported that rottlerin blocks glycogen synthase kinase 3β, malate dehydrogenase, and activated calcium-activated K^+^ channel (Soltoff, 2007). Collectively, insufficient inhibitors selectively affect different PKC isoforms simultaneously. Different PKC isoforms have complex interactions and regulation, which may display antagonistic effects in these studies.

Given the critical roles of PKC isoforms in the progression of diabetes-associated atherosclerosis, further *in vitro* and *in vivo* animal studies and clinical trials in humans will be necessary to define the specific functions of PKC in diabetes-associated atherosclerosis. Large-scale screening of isoform-specific PKC inhibitors is necessary to identify the key enzyme at each phase of diabetes-associated atherosclerosis and to understand the functional interplay between the isoforms. The next step in PKC research in diabetes-associated atherosclerosis is to expand the *in vivo* investigations. Approaches such as transplantation of bone marrow from isoform-specific null mice to atherosclerosis-prone mouse models (*Ldlr* ^*−/−*^ or *ApoE* ^*−/−*^ mice) and generation of double knockout mice may help to evaluate the functional role of each PKC isoform during the progression of diabetes-associated atherosclerosis. A combination of these genetic approaches and highly selective pharmacological tools will facilitate in confirming the role of PKC in diabetic atherosclerosis and potentially assist the search for clinical therapeutics.

## Conclusion

Our review indicated that PKC plays an extensive role in the pathophysiology of diabetes and atherosclerosis, and therefore could be an excellent therapeutic target for stage-specific diabetes-associated atherosclerosis. To identify the key PKC isoforms involved in the pathogenesis and to determine whether the combinations of isoform-specific inhibitors might have synergistic effects in the treatment of diabetes-associated atherosclerosis are two potential challenges in developing therapeutic agents targeting PKC for ASCVD. Though further confirmatory *in vitro* and *in vivo* studies, and human studies are required, we anticipate that the promising role of PKC can potentiate the treatment of diabetes-associated atherosclerosis in humans.
